# Effects of ammonia on growth performance, lipid metabolism and cecal microbial community of rabbits

**DOI:** 10.1371/journal.pone.0252065

**Published:** 2021-06-30

**Authors:** Jia Cui, Xinyu Yang, Fengxia Wang, Shudong Liu, Shuaijuan Han, Baojiang Chen

**Affiliations:** College of Animal Science and Technology, Hebei Agricultural University, Bao ding, China; Tokat Gaziosmanpasa Universitesi, TURKEY

## Abstract

This study was designed to investigate the effect of ammonia on growth performance, lipid metabolism and intestinal flora of rabbits. A total of 150 female IRA rabbits (35-days-old) were randomly divided into three groups including 0 ppm (CG), 10 ppm (LAC) and 30 ppm ammonia (HAC) groups for a period of 28 days. The average daily weight gain (ADG) of rabbits was significantly reduced in LAC (-17.11%; *p* < 0.001) and HAC groups (-17.46%; *p* < 0.001) as compared with the CG. Serum concentration of high density lipoprotein (HDL) and glucose (Glu) were increased in LAC (+80.95%; +45.99; *p* < 0.05) and HAC groups (+219.05%; +45.89; *p* < 0.001), while apolipoprotein A1 (apoA1) was decreased in LAC (-58.49%; *p* < 0.001) and HAC groups (-36.92%; *p* < 0.001). The structural integrity of cecum was damaged, and the thickness of mucosa and serosa were significantly decreased in LAC and HAC. The acetate, butyrate and propionate level of cecal chyme were reduced in HAC group (-21.67%; -19.82%; -30.81%; *p* < 0.05). Microbial diversity and burden of Firmicutes were significantly decreased, while that of pathogenic bacteria, such as Bacteroidetes, Clostridium and Proteobacteria were increased in ammonia treated groups. Spearman’s correlation confirmed that burden of Ruminococcaceae_NK4A214_group showed significantly negative correlation with acetic acid (r = -0.67; *p* < 0.001) while Barnesiellaceae_unclassified showed significantly positive correlation with propionic acid (r = 0.50; *p* < 0.001). In conclusion, ammonia treatment was responsible for an imbalance of intestinal flora, which affected lipid metabolism and damaged intestinal barrier of rabbits, resulting in low growth performance due to lipid metabolism dysfunction.

## 1. Introduction

Rabbit is now considered as one of the popular farmed animals because of its short feeding cycle and easy management. Although rabbits are now kept for breeding purpose on a large scale, still the traditional courtyard and small-scale breeding modes of rabbits keeping are the dominant one in China [1]. With increase in breeding density, the breeding environment of rabbit farms is getting worst, for which an obvious indicator is an increase of ammonia concentration in the environment [2]. Rabbits are particularly sensitive to air quality with high ammonia concentration which significantly increases the incidence of respiratory (such as infectious rhinitis, etc.) as well as eye infections in rabbits [3]. Previous studies have confirmed that high-concentration of ammonia affects the respiratory, nervous, immune and reproductive systems [4–12]. Recently, very few reports have been established on the harmful effects of ammonia on rabbits, and Environmental Quality Standards for Livestock and Poultry Farms (NY/T388) has not yet specified the maximum tolerated limit of ammonia for rabbit house.

High level of ammonia can trigger chronic liver insufficiency, oxidative stress, intestinal immunity and morphology [13, 14]. High ammonia concentration damages jejunal integrity which causes an abnormal loss of jejunal microvilli [15, 16], changes cecal flora of laying ducks, and thus results in abnormal lipid metabolism [17, 18]. Exposure to high NH_3_ concentration regulate fat distribution in broilers [19] affecting the activities of key fat synthesis and decomposition enzymes, expression of related genes and proteins, and pathways related to lipid metabolism [20], thereby regulating body lipid metabolism [21]. In ileum, *Blautia*, *Bifidobacterium*, *Subdoligranulumum* and *Coprococcus 3* are observed to have positive correlation with lipid metabolism dysfunction and pro-inflammatory responses in low birth weight pigs [22].

The cecum has an important position in the digestive system having abundant microbial communities which are responsible for both the digestion and absorption of nutrients [23]. Microbial flora colonized in cecum is resistant to foreign strains and thus inhibit invasion and colonization of pathogens [24]. The microbial flora, intestinal epithelial mucosa, intestinal mucus and digestive juice are the weapons of intestinal barrier [25]. The microbial flora can also have a role in the regulation of immune system by controlling epithelial cells proliferation and differentiation to protect the health status of animals [26]. Therefore, structural integrity and floral stability of cecum are essential for rabbit’s health because of their involvement in the production performance of rabbits [17].

Up to date, information regarding the influence of different concentrations of ammonia on intestinal micro-flora, lipid metabolism and its correlation in rabbits, still needs to be elucidated. Therefore, this study was designed to investigate the effects of ammonia on growth performance, lipid metabolism and intestinal flora of rabbits.

## 2. Materials and methods

### 2.1. Ethics statement

All experiments were performed in accordance with the ARRIVE guidelines and were carried out in accordance with the U.K. Animals (Scientific Procedures) Act, 1986 and associated guidelines, EU Directive 2010/63/EU for animal experiments, and the National Institutes of Health guide for the care and use of Laboratory animals (NIH Publications No. 8023, revised 1978), approved by the Ethics Committee of Animal Experimentation of Hebei Agricultural University (Protocol 2021083).

We assessed animals’ status four times a day including the feed and water consumption, environmental NH_3_ level and animal behavior. We referred to the Guideline of assessment for humane endpoints in animal experiment (RB/T 173–2018) http://www.lascn.net/Item/75841.aspx.

The item includes four main criteria that would be monitored: body weight, general condition, physiological indicators and spontaneous behavior. Body weight: body weight loss > 20% or refusal eat and drink; General condition: extremely messy and dirty coat; abnormal posture; dull eyes; severe respiratory infection, breathing difficulty; Physiological indicators: body temperature changes > 2°C, accelerate or decrease heart rate or breathing rhythm by more than 50%; Spontaneous behavior: abnormal behavior, reduced motor activity or hyperkinesia, not responsive, psychomotor agitation.

### 2.2. Animals, management and experimental diets

A total of 150 IRA female rabbits (35-days-old) were purchased from Xingtai Kangming Co., Ltd., Xingtai, China, which were randomly divided into 3 groups named as control group (CG), low ammonia concentration (LAC) and high ammonia concentration (HAC) groups with 0, 10 and 30 ppm of ammonia, respectively. Each group was further divided into 5 replicates with 10 rabbits per replicate which were housed in wire cages in an environmentally controlled house with desired ammonia concentrations (0, 10 and 30 ppm). The three chambers were identical in terms of size [23.5 × 23.5 × 2.5 m (length × width × height)], construction materials, acclimatization equipment, cages, feeders and drinkers, commercially prepared by Zhejiang Qiushi Artificial Environment Co., Ltd., Zhejiang, China. The H_2_S and CO_2_ concentrations were maintained at < 0.2 mg/m^3^ and < 1500 mg/m^3^, respectively. The ammonia bottles were connected with pressure regulator and flow meter to maintain the ammonia level. When the ammonia concentration was exceeded from the set concentration, it exhausted the air until reached the set concentration. No significant difference was observed in the initial live weight of rabbits, access to feed and water was *ad libitum*. The chamber temperature was maintained between 18 and 24°C and moisture contents between 60% and 70% with 12 h of light and dark cycles. In addition, rabbits were vaccinated against rabbit plague. The basal diet for rabbits was prepared according to the recommendations of National Research Council (NRC) [27]. The feed composition is provided in Table 1.

**Table 1:** Chemical composition levels of basal diets (air-dried basis) %.

Ingredients	Percent (%)	Index	Nutrient levels
Corn	16.50	DM	89.01
Wheat bran	10.00	DE/(MJ/kg)	9.20
Soybean meal	16.00	CP	17.14
Alfalfa meal	26.00	EE	3.21
Oat meal	27.50	CF	13.99
NaCl	0.50	NDF	35.24
Lys	0.08	ADF	17.30
Met	0.12		
Zeolite meal	1.00		
Premix^a^	0.30		
Palm oil meal	0.50		
Bentonite	1.50		
Total	100.00		

a. The premix provided per kg of the diet: Fe (as ferric sulfate) 70 mg, Cu (as copper sulfate) 20 mg, Zn (as zinc sulfate) 70 mg, Mn (as manganese sulfate) 10 mg, Se (as sodium sulfate) 0.25 mg, Co 0.15 mg, I 0.2 mg, VA 10 000 IU, VD 900 IU, VE 50 mg, VK 2 mg, VB_1_ 2 mg, VB_2_ 6 mg, VB_12_ 0.02 mg, Pantothenic acid 50 mg, Pyridoxine 2 mg, Nicotinic acid 50 mg, choline 1 000 mg, Biotin 0.2 mg.

### 2.3. Growth performance and sample collection

The feed intake and live weight of each replicate were recorded on weekly basis and the average daily feed intake (ADFI), average daily gain (ADG) and feed conversion ratio (FCR) were calculated accordingly. On the 28^th^ day of trial, six rabbits from each replicate (30 rabbits per group) were randomly selected and euthanized by professional veterinarians who has the Practicing veterinary certificate according to AVMA (American Veterinary Medical Association) [28]. Sodium pentobarbital was used as anesthetic and was administered intravenously. Blood samples were rapidly collected in serum separation tubes, centrifuged at 3000 rpm at 4°C for 15 min, and stored at -20°C till further analysis. Spleen and liver were collected and organ index was calculated. The cecal digesta was immediately frozen in liquid nitrogen at -80°C till DNA extraction.


Anorganindex=Theweightofanorgan(g)/Theweightofabody(kg)×100%

### 2.4. Histological examination

Six histological sections were obtained from cecal tissues of each group. Routine H&E-stained sections were cut at approximately 3- to 4-um thickness after fixation (formalin) and paraffin embedding. The cut paraffin tissue sections were put in hot water bath to separate paraffin wax, and tissue sections were mounted on gelatin-coated slides, dried, de-waxed, stained with hematoxylin & eosin (H&E) and sealed. Structural changes in the cecal tissues were examined under a microscope (CKX41).

### 2.5. Concentrations of lipid metabolism indexes in the serum of the rabbits

Total cholesterol (TC), triglyceride (TG), high density lipoprotein (HDL), low density lipoprotein (LDL), glucose (Glu), apolipoprotein A1 (apoA1), apolipoprotein B (apoB) and insulin contents were tested from the collected rabbit serum using competitive enzyme-linked immunosorbent assay (ELISA) kits (BaiZhi Bio-Engineering Technology Company, Beijing, China) following manufacturer’s instructions.

### 2.6. Short chain fatty acid (SCFA)

Cecal contents of each group were obtained in six tubes and were labeled accordingly. The short chain fatty acids namely acetate, propionate and butyrate were measured from the total bile acid concentration in freeze-dried feces using an enzymatic reagent kit (IDK Bile Acids Photometric test; Immundiagnostik, Bensheim, Germany).

### 2.7. Intestinal microbiome analysis

Following manufacturer’s protocol, the Bacterial Genomic DNA (gDNA) was extracted from samples using Power Soil DNA Isolation Kit (Omega Bio-Tek Inc., Norcross, GA, USA). The quality and quantity of DNA were assessed by 260 nm/280 nm and 260 nm/230 nm ratios and stored at -80°C till further analysis.

Through polymerase chain reaction (PCR), the V3-V4 bacterial region of 16S rRNA gene was amplified using 338-F (5’ ACTCCTACGGAGG CAGCA -3’) and 806-R (5’-GGACTACHVGGGTWTCTAAT-3’) primers combined with adapter and barcode sequences. A total of 50 μL PCR mixture containing buffer and high GC enhancer (10 μL each), Q5 high-fidelity DNA polymerase (0.2 μL), dNTP (1 μL), primer (10 μM each) and gDNA (60 ng) was prepared. The PCR was performed as: an initial denaturation (95°C for 5 min), followed by 15 cycles (95°C for 1 min, 50°C for 1 min and 72°C for 1 min) and final extension (72°C for 7 min). These first step PCR products were purified using VAHTS™ DNA Clean Beads (Vazyme Biotech Co., Ltd., China). The second step of PCR was then performed with a 40 μL PCR mixture containing 2× Phμsion HF MM (20 μL), ddH2O (8 μL), primer (10 μM each) and first step PCR products (10 μL). The PCR conditions were as follows: an initial denaturation (98°C for 30 s), followed by 10 cycles (98°C for 10 s, 65°C for 30 s and 72°C for 30 s) and final extension (72°C for 5 min). All PCR products were quantified by Quant-iT™ dsDNA HS reagent (Calbiochem Co., Ltd., Germany) and pooled together. High-throughput sequencing analysis of bacterial rRNA gene was done using the Illumina Hiseq 2500 platform (2 × 250 paired ends) at Biomarker Technologies Corporation, Beijing, China.

According to the relationship between paired-end (PE) reads and overlapping reads, the double-ended sequencing data was compiled into a sequence of tags after Hiseq sequencing. The quality of the reads and the effect of merging analyzed by quality control were used to obtain valid data by three steps method (including PE read splicing, tag filtering and the removal of chimerism). UCLUST was used in QIIME (version 1.8.0) software to cluster tags at a similarity level of 97% to obtain operational taxon unit (OTUs) and classify the OTUs based on Silva taxonomy database (https://www.arb-silva.de/)

### 2.8. Statistical analysis

Statistical Package for the Social Sciences (SPSS) 19.0 software (SPSS Inc., Chicago, IL, USA) was used for statistical analyses. One-way analysis of variance (ANOVA) and Duncan’s post-tests were performed to calculate the differences in growth performance, serum lipid metabolism, alpha diversity and bacterial taxa abundance at the phylum and genus levels among three groups. The results were expressed as Mean ± Standard Deviation (SD). *p* < 0.05 was considered a significant difference. *p* < 0.01 was considered a high significant difference. GraphPad-Prism version 7.0 (San-Diego, CA, USA) and heatmap illustrator (HemI, version 1.0.3.7) software’s were used to drawn the graphs and Heatmap, respectively.

## 3. Results

### 3.1. Effects of ammonia on growth performance of rabbits

As shown in Table 2, compared with the CG group, the final weight (FW) and ADG of rabbits were significantly decreased while FCR was significantly higher in HAC and LAC groups. Spleen index (SI) was significantly lowered in HAC group (-25.58%; *p* < 0.05) compared with the CG. However, among these three groups, no significant difference was observed in ADFI and liver index (LI).

**Table 2:** Effects of ammonia on growth performance of rabbits.

	CG	LAC	HAC	*p* -value
IW(g)	1300.59±140.12	1336.05±118.09	1334.6±145.88	0.39
FW (g)	2580.07±199.11^a^	2392.08±213.87^b^	2395.33±162.62^b^	<0.001
ADG (g/d)	45.70±6.88	37.88±6.00	37.72±6.52	<0.001
ADFI (g/d)	157.35±23.43^b^	161.02±35.90^a^	150.05±32.26^c^	0.27
FCR (g/d)	3.46±0.38^c^	4.25±0.63^a^	3.97±0.45^b^	<0.001
LI (g/kg)	18.35±3.07	16.07±2.00	16.34±1.73	<0.01
SI (g/kg)	0.43±0.074^a^	0.42±0.12^a^	0.32±0.068^b^	0.05

^a, b^ Different superscript letters within a row indicate significant differences at *p* < 0.05. CG, control group (0 ppm); LAC, low ammonia concentration (10 ppm); HAC, high ammonia concentration (30 ppm). IW: initial weight, FW: final weight, ADG: average daily gain, ADFI: average daily feed intake, FCR: feed conversion ratio, LI: liver index, SI: spleen index.

### 3.2. Concentrations of lipid metabolism indexes in rabbits serum

As shown in Table 3, the TC and insulin level were not affected by any of the ammonia treatments. Compared with CG, the content of LDL and Glu were increased in LAC (+42.11%; +45.99; *p* < 0.001) and HAC groups (+23.68%; +45.89; *p* < 0.001), while apoA1 was decreased in LAC (-58.49%; *p* < 0.001) and HAC groups (-36.92%; *p* < 0.001).

**Table 3:** Effects of ammonia on lipid metabolism indexes in rabbit’s serum.

	CG	LAC	HAC	*p*-value
TC (mM)	0.95±0.13	0.86±0.082	0.85±0.071	0.05
TG (mM)	0.69±0.081^a^	0.62±0.071^a^^b^	0.46±0.20^b^	0.29
HDL (mM)	0.21±0.068^c^	0.38±0.063^b^	0.54±0.07^a^	0.02
LDL (mM)	0.38±0.034^b^	0.54±0.10^a^	0.47±0.048^a^^b^	<0.001
Glu (mg/dL)	100.36±7.73^b^	146.52±14.82^a^	146.42±15.41^a^	<0.001
apoA1 (ug/ml)	1235.41±148.93^a^	512.82±130.31^c^	779.25±161.22^a^	<0.001
apoB (ug/ml)	18.53±3.49^b^	31.23±7.47^a^	25.88±10.04^a^^b^	0.09
insulin (pmol/L)	18.25±5.44	18.12±7.90	15.14±5.96	0.72

Values are means ± SD for n = 6 rabbits per group.

^a, b^ Different superscript letters within a row indicate significant differences at *p* < 0.05. CG, control group (0 ppm of ammonia); LAC, low ammonia concentration (10 ppm); HAC, high ammonia concentration (30 ppm). TC: Total cholesterol, TG: triglyceride, HDL: high density lipoprotein, LDL: low density lipoprotein, Glu: glucose, apoA1: apolipoprotein A1, apoB: apolipoprotein B.

### 3.3. Short-chain fatty acids concentration in the cecal digesta of rabbits

**Fig 1:**
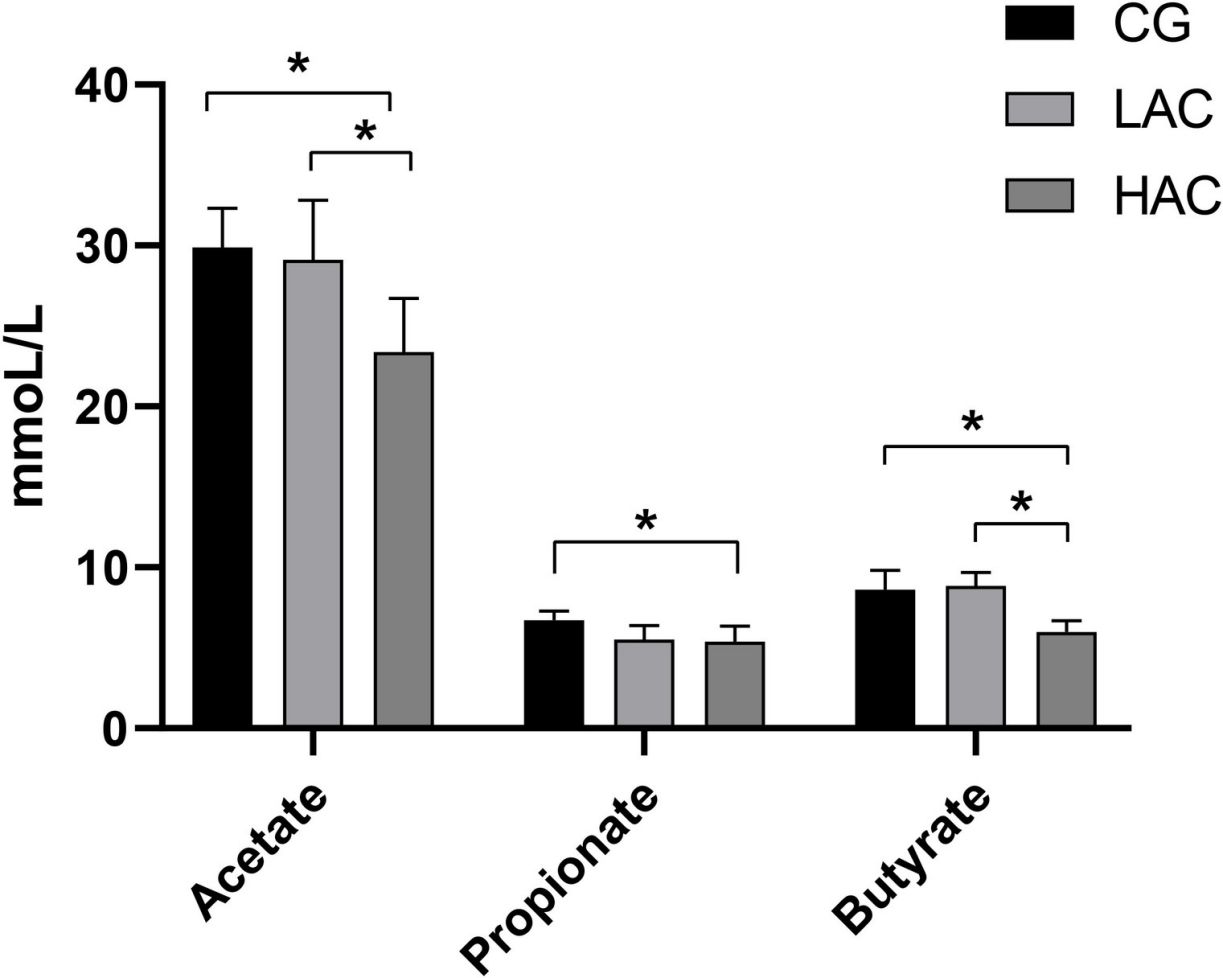
The concentrations of acetate, butyrate and propionate in cecal digesta. * *p* < 0.05 and ** *p* < 0.01 indicates significant and high significant differences, respectively. CG, control group (0 ppm; LAC, low ammonia concentration (10 ppm); HAC, high ammonia concentration (30 ppm).

Compared with CG, acetate and butyrate were decreased significantly in LAC and HAC (Fig 1), while propionate was decreased in HAC group only.

### 3.4. Effect of ammonia on cecal morphology

**Fig 2:**
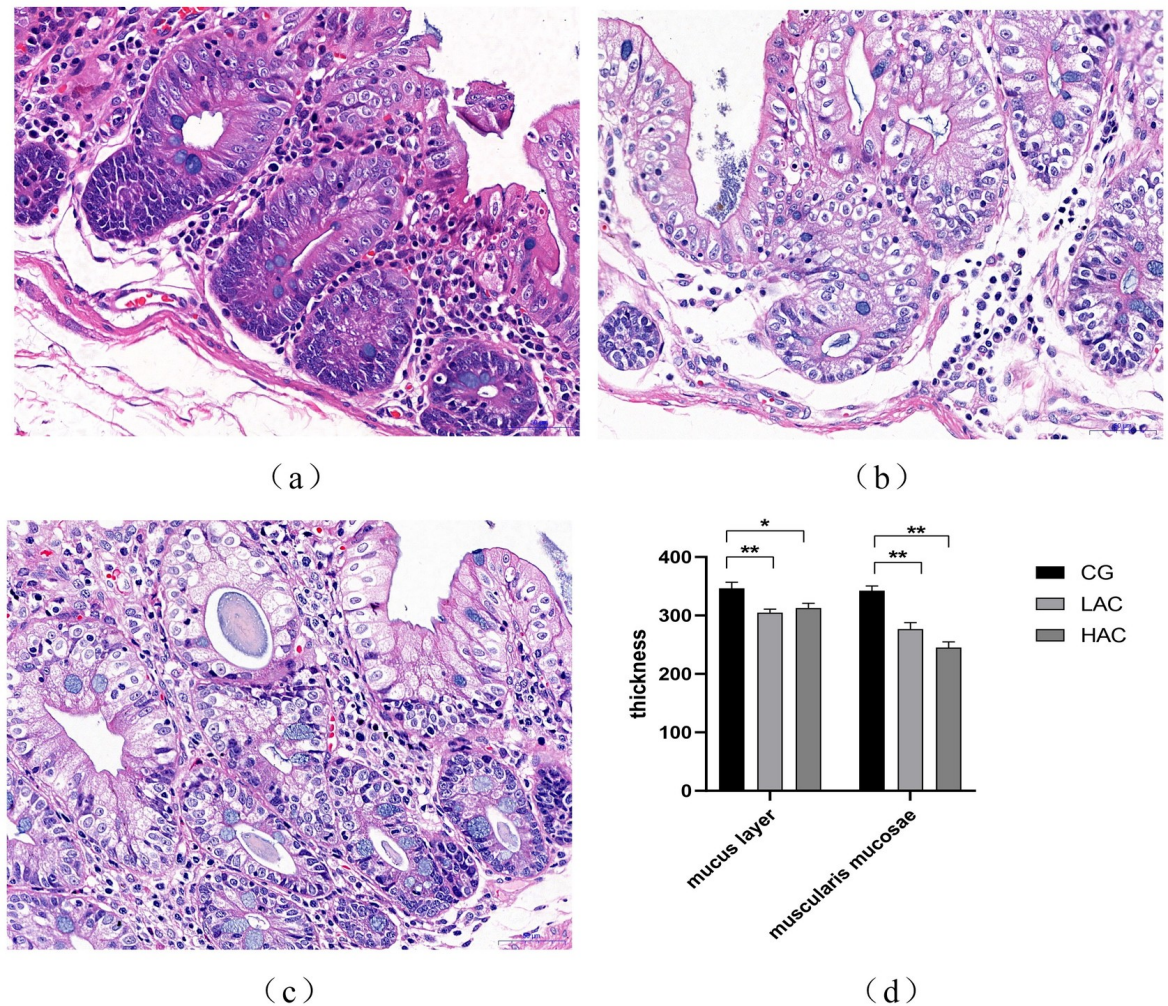
Analysis of cecal morphology between CG, LAC and HAC groups. a: cecal shape in CG (×400); b: cecal shape in LAC group (×400); c: cecal shape in HAC group (×400); d: count of mucosal and serosal thickness. The vacuolar degeneration of mucosal epithelium is represented by a green arrow (↑), lymphocytes by an orange arrow (↑), and plasma cells by a red arrow (↑). *^,^ ** indicate significant (*p* < 0.05) and highly significant (*p* < 0.01) differences, respectively. CG, control group (0 ppm); LAC, low ammonia concentration (10 ppm); HAC, high ammonia concentration (30 ppm) groups.

The histopathological observations revealed that high concentration of ammonia destroyed the structural integrity of cecum. In LAC and HAC groups, vacuolar degeneration of mucosal epithelial cells in the cecal tissues and edema of lamina propria were observed (Fig 2B and 2C). The thickness of cecal mucosa and muscle membrane were significantly reduced (*p* < 0.05; Fig 2D).

### 3.5. Effects of ammonia on cecal microbial diversity

As shown in Table 4, the alpha diversity of cecal microbiota of rabbits was influenced by ammonia treatment. Simpson of cecal microbial diversity was higher in the HAC group (+1.01%; *p* < 0.01) than that in the CG. However, Shannon and Chao1 microbial diversity indices were non significantly altered by ammonia.

**Table 4:** Alpha diversity analysis of the cecal microbiota of rabbits.

Group	CG	LAC	HAC	*p*-value
Observed otus	637.17±52.45	714.83±70.25	752.17±50.28	0.39
Shannon	8.00±0.10	8.03±0.11	7.75±0.15	0.27
Simpson	0.99±0.00^a^	0.99±0.00^a^	0.98±0.0031^b^	<0.01
Chao1	640.88±53.67	720.70±72.42	756.55±51.07	0.40

Values are means ± SD for n = 6 rabbits per group.

^a, b^ Different superscript letters within a row indicate significant differences at *p* < 0.05. CG, control group (0 ppm); LAC, low ammonia concentration (10 ppm); HAC, high ammonia concentration (30 ppm).

**Fig 3:**
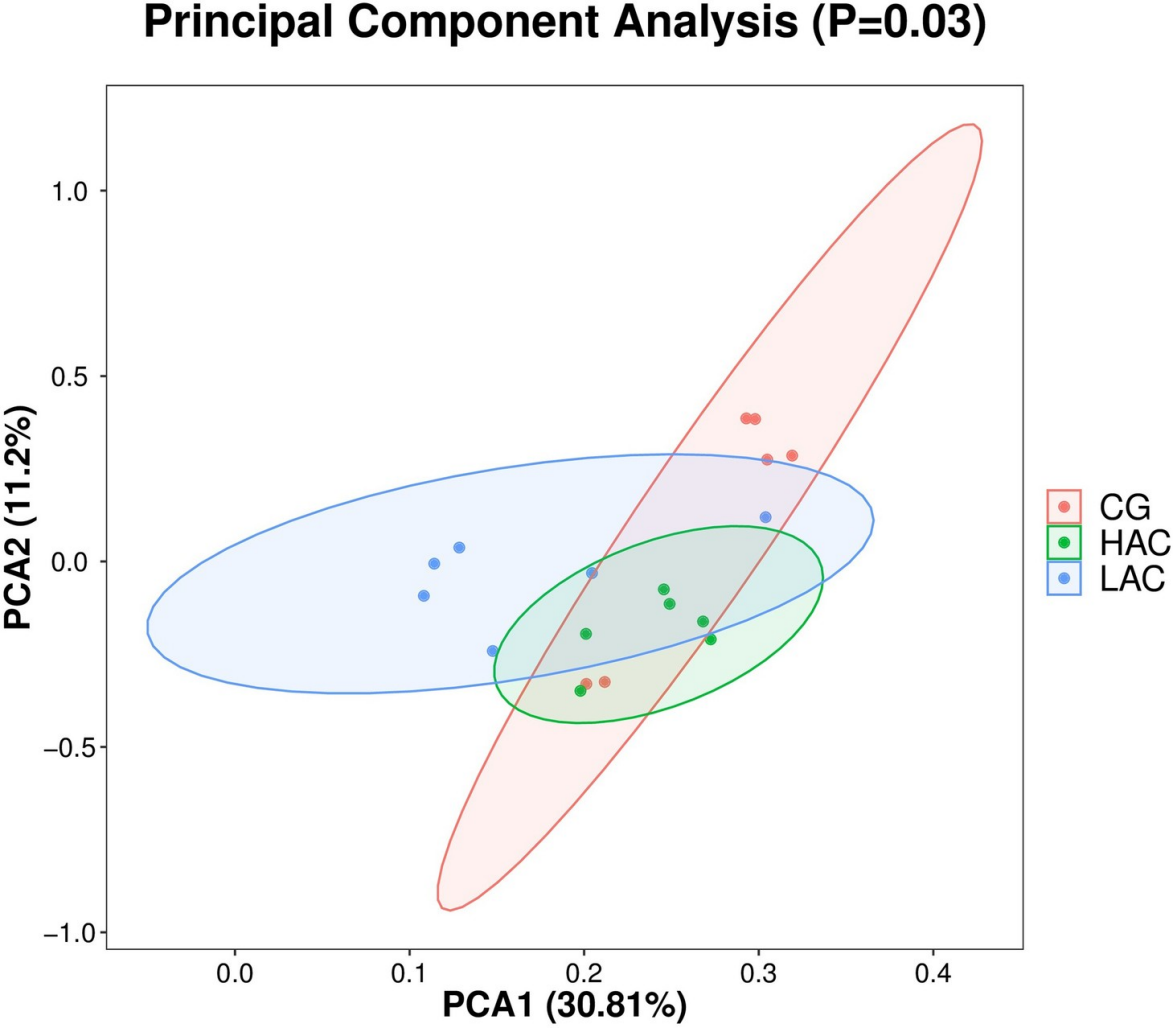
Beta diversity analysis of microbial communities using principal component analysis (PCA) based on un-weighted UniFrac distances. CG, control group (0 ppm); LAC, low ammonia concentration (10 ppm); HAC, high ammonia concentration (30 ppm).

The cecal microbiota samples from LAC group were clustered together and clearly separated from the cecal microbiota from CG and HAC groups as shown in Fig 3.

### 3.6. Effects of ammonia on cecal microbial composition

**Fig 4:**
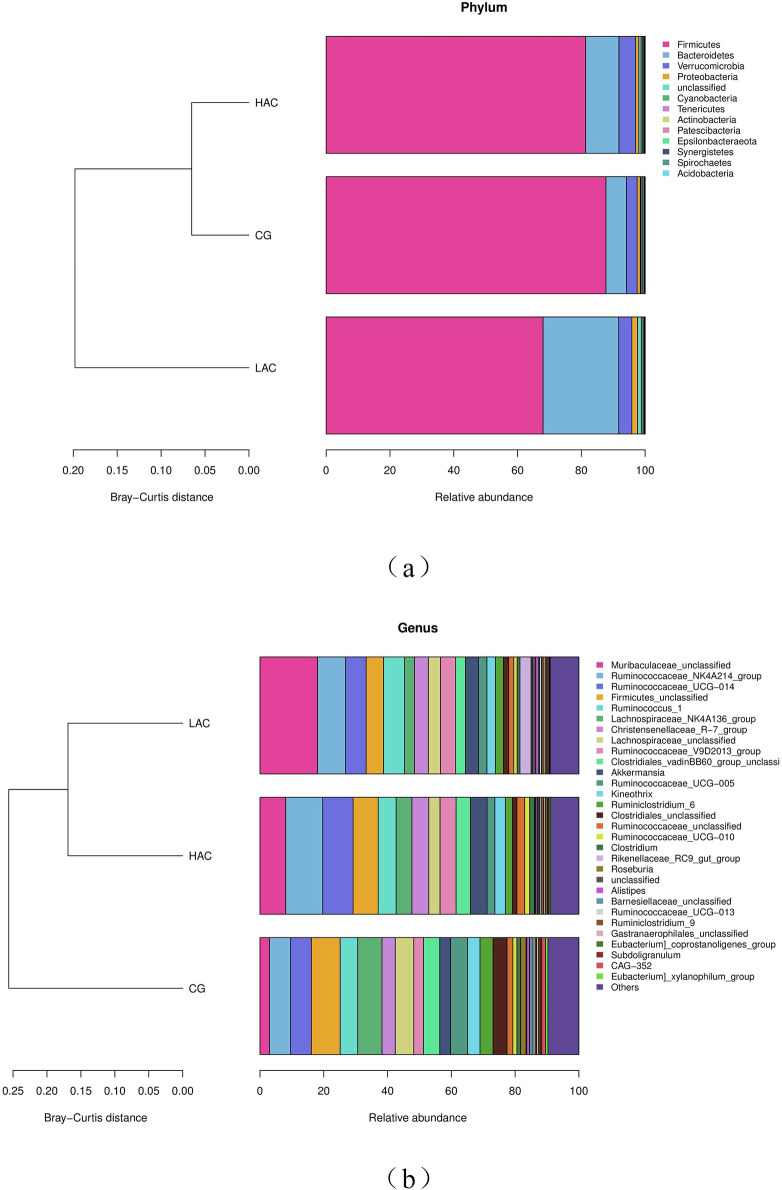
The relative burden of bacterial phyla (a) and genera (b) in the cecal microbiota of rabbits in different groups. CG, control group (0 ppm); LAC, low ammonia concentration (10 ppm); HAC, high ammonia concentration (30 ppm).

We compared the microbial communities at phylum and genus levels. The predominant phyla were Firmicutes, Bacteroidetes and Verrucomicrobia, accounted as 97.5%, 95.83% and 97.03% for CG, LAC and HAC groups, respectively in the cecal digesta of rabbits (Fig 4 and Table 5). Compared with the CG, ammonia decreased the relative burden of Firmicutes in the LAC (-22.49%; *p* < 0.01) and HAC groups (-7.26%; *p* < 0.01). The genus belongs to Firmicutes, such as *Lachnospiraceae_NK4A136_group*, *Lachnospiraceae_unclassed* and *Clostridiales_vadinBB60_group_unclassed* were decreased. However, the relative abundance of Bacteroidetes was higher in the LAC (+269.62%; *p* < 0.01) and HAC groups (+62.62%; *p* < 0.01) compared with the CG. Its genus such as *Muribaculaceae_unclassified* was also increased. No significant differences were observed in the relative burden of other phyla between the three groups.

**Table 5:** Differentially abundant phyla in the cecum of rabbits of three treatments.

Phylum	CG	LAC	HAC	*p*-value
Firmicutes	87.74±4.58^a^	68.01±10.92^c^	81.37±8.45^b^	<0.01
Bacteroidetes	6.42±2.28^c^	23.73±10.60^a^	10.44±8.17^b^	<0.01
Verrucomicrobia	3.34±2.22	4.10±3.40	5.22±2.39	0.50
Proteobacteria	0.98±0.87	1.82±2.36	1.01±0.42	0.55
Unclassified	0.25±0.15	1.14±1.11	0.69±0.63	0.15
Cyanobacteria	0.59±0.47	0.58±0.68	0.52±0.25	0.97
Tenericutes	0.42±0.37	0.37±0.51	0.34±0.46	0.95
Actinobacteria	0.15±0.088	0.15±0.10	0.19±0.062	0.66
Patescibacteria	0.073±0.061	0.05±0.050	0.17±0.16	0.15
Epsilonbacteraeota	0.047±0.028	0.053±0.032	0.032±0.026	0.55

Values are means ± SD for n = 6 rabbits per group.

^a, b^ Different superscript letters within a row indicate significant differences at *p* < 0.05. CG, control group (0 ppm); LAC, low ammonia concentration (10 ppm); HAC, high ammonia concentration (30 ppm).

### 3.7. Function prediction STAMP difference analysis

**Fig 5:**
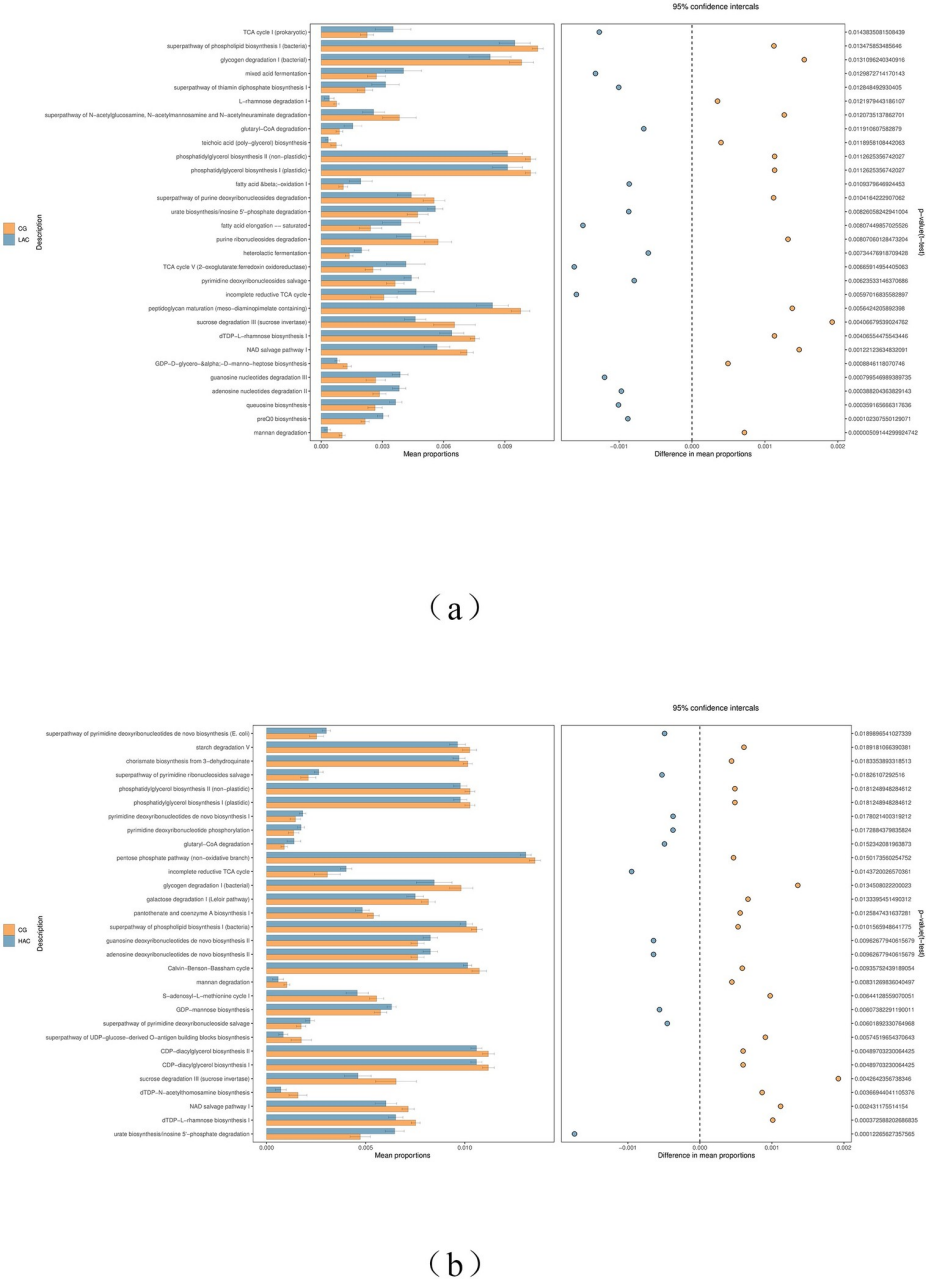
Predicted microbial function comparison. (a) Comparison of cecal microbial function between 10 ppm and (b) 30 ppm ammonia (n = 6) and control (n = 6) groups. Statistical analyses were conducted by two-sided Welch’s t-test and Benjamini–Hochberg FDR correction between two groups, and the *p*-value of different functions lower than 0.05 was shown.

We analyzed transporters pathway of cecal microbiota using the pathway terms (Fig 5A–5B). Super pathway of phospholipid biosynthesis I (bacterial), glycogen degradation I (bacterial), phosphatidylglycerol biosynthesis II (non-plastidic), phosphatidylglycerol phosphate biosynthesis I (plastidic), sucrose degradation III (sucrose invertase), NAD salvage pathway I and mannan degradation pathway were significantly decreased in the ammonia treated groups.

### 3.8. Potential pathogenic prediction

**Fig 6:**
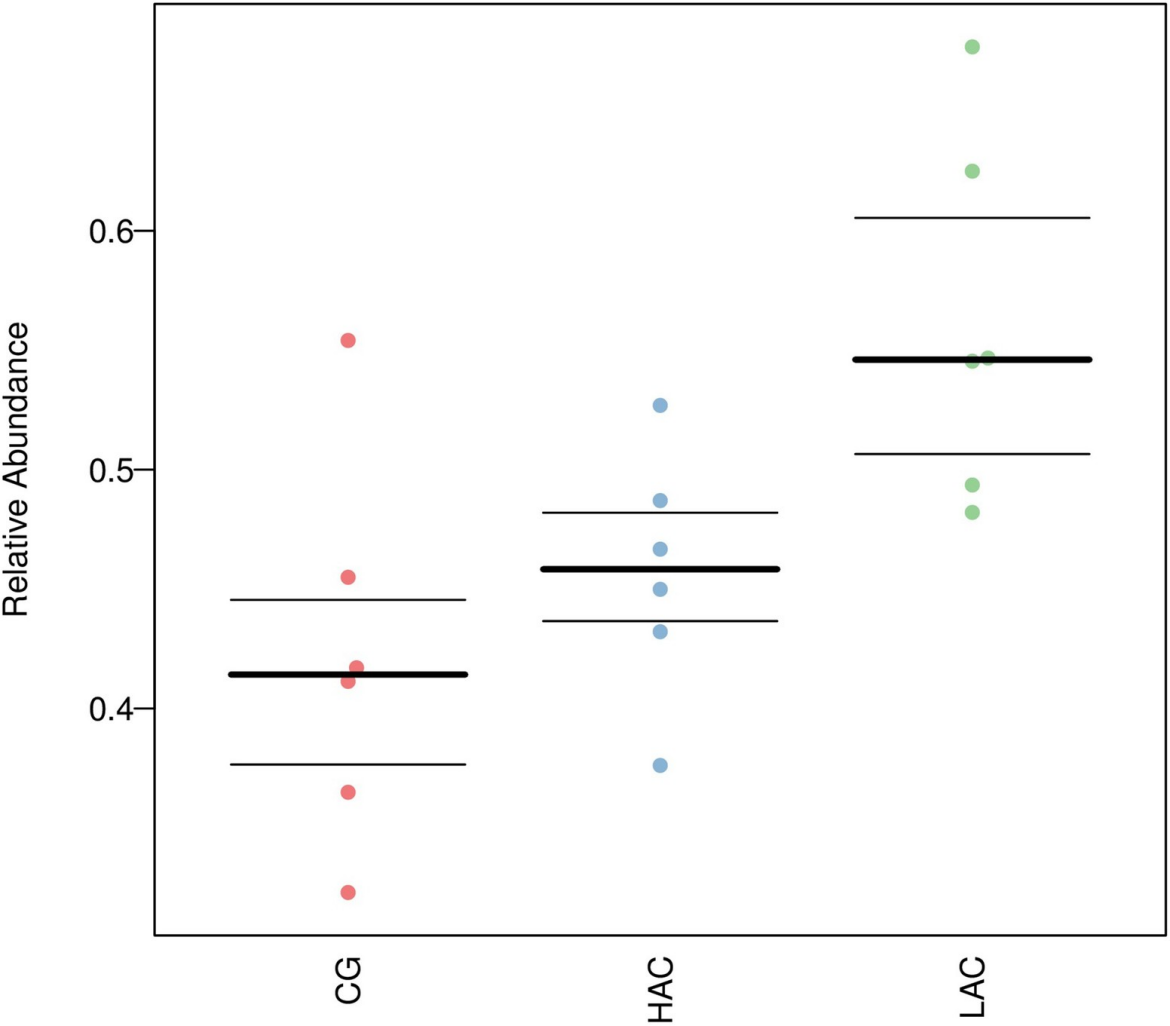
Prediction of potentially pathogenic bacteria. CG, control group (0 ppm); LAC, low ammonia concentration (10 ppm); HAC, high ammonia concentration (30 ppm).

As shown in Table 6 and Fig 6, potential pathogenic bacteria were increased after ammonia treatment.

**Table 6:** Differentially abundant genera in the cecum of rabbits of three treatments.

Genus	CG	LAC	HAC	*p*-value
*Muribaculaceae_unclassified*	2.97±2.02^b^	18.05±13.37^a^	8.09±8.12^a^^b^	0.03
*Ruminococcaceae_NK4A214_group*	6.65±1.11^b^	8.81±3.88^a^^b^	11.60±3.59^a^	0.05
*Lachnospiraceae_NK4A136_group*	7.61±1.60^a^	3.1±2.41^b^	4.95±3.46^a^^b^	0.03
*Ruminococcaceae_UCG-005*	5.37±1.37^a^	2.65±0.57^b^	2.45±0.49^b^	0.07
*Clostridium*	1.15±0.23^a^^b^	0.90±0.063^b^	1.50±0.55^a^	0.11
*Roseburia*	1.72±0.71^a^	0.33±0.54^b^	0.31±0.25^b^	0.06
*Barnesiellaceae_unclassified*	1.10±0.13^a^	0.41±0.18^b^	0.31±0.090^b^	<0.01
*CAG-352*	1.19±0.42^a^	0.11±0.059^b^	0.14±0.11^b^	0.01
*Eubacterium]_xylanophilum_group*	0.76±0.15^a^	0.21±0.057^b^	0.46±0.15^b^	0.03

Values are means ± SD for n = 6 rabbits per group.

^a, b^ Different superscript letters within a row indicate significant differences at *p* < 0.05. CG, control group (0 ppm); LAC, low ammonia concentration (10 ppm); HAC, high ammonia concentration (30 ppm).

### 3.9. Correlation analysis of altered cecal bacteria with serum lipid metabolism indexes and SCFA

**Fig 7:**
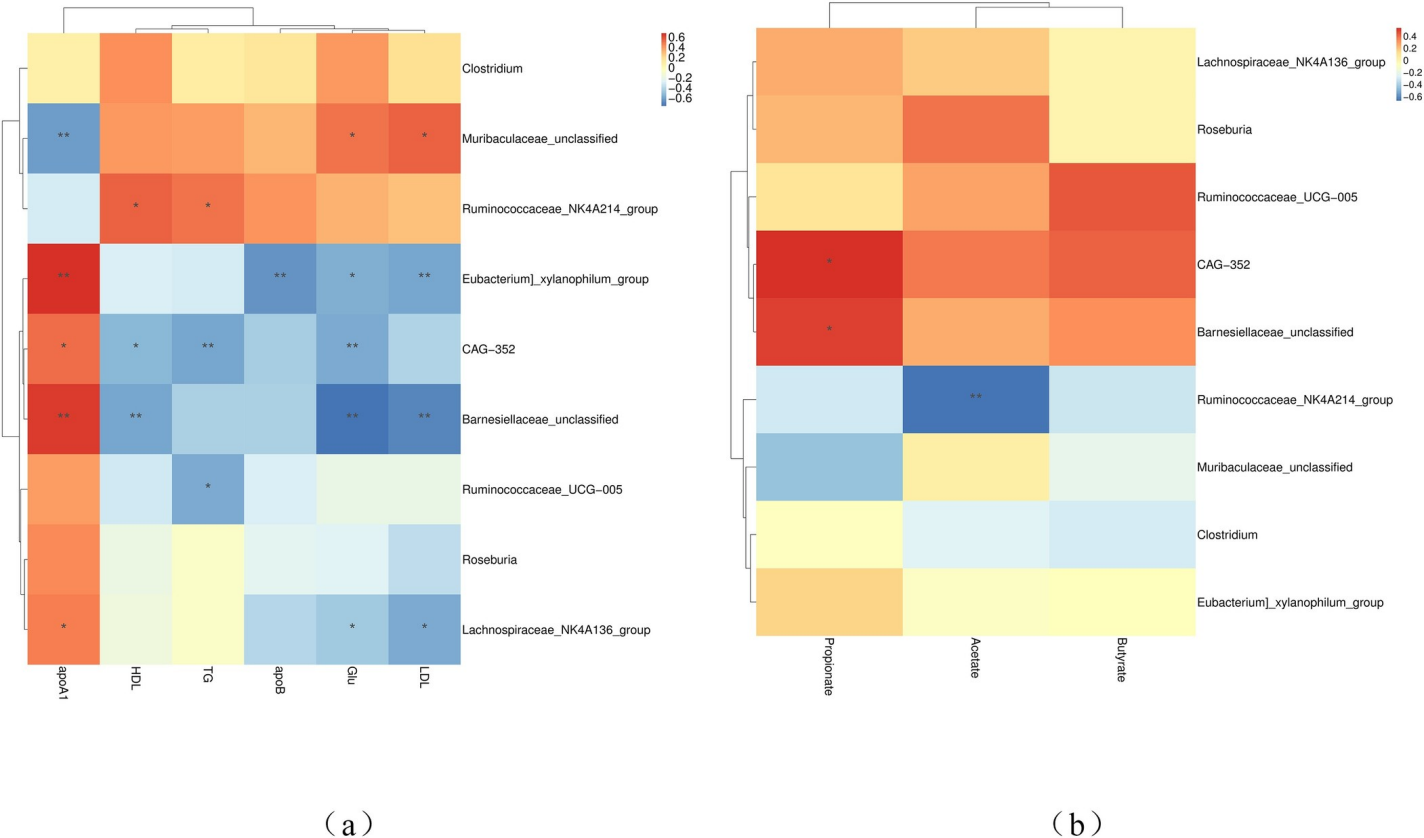
Spearman’s rank correlation analysis (SRCA) between significantly modified microbiota, serum lipid metabolism indexes and SCFA concentrations of rabbits. * indicates *p* < 0.05 and ** indicates *p* < 0.01. Red and blue colors represent positive and negative correlations, respectively. CG, control group (0 ppm); LAC, low ammonia concentration (10 ppm); HAC, high ammonia concentration (30 ppm); TC, total cholesterol; TG, triglyceride; HDL, high density lipoprotein; LDL, low density lipoprotein; glucose, Glu; apoA1, apolipoprotein A1; apoB, apolipoprotein B; ins, insulin.

Correlation analysis revealed that burden of genus *Muribaculaceae_unclassified* showed a strong positive correlation with LDL and Glu and a negative correlation with apoA1 (Fig 7). The abundance of genus *Ruminococcaceae_NK4A214_group* showed positive correlation with TG and HDL, and negative correlation with acetate. The genus *Lachnospiraceae_NK4A136_group* showed strong positive correlation with apoA1 and strong negative correlation with LDL and Glu. The abundance of genus *Ruminococcaceae_UCG-005* was negatively correlated with TG. The burden of genus *Barnesiellaceae_unclassified* was positively correlated with apoA1 and propionate, and negatively correlated with HDL, LDL and Glu. The abundance of genus *CAG-352* was positively correlated with apoA1 and acetate, and negatively correlated with TG, HDL and Glu. In addition, the burden of genus *Eubacterium_xylanophilum_group* showed a strong positive correlation with apoA1 and negative correlation with LDL and Glu.

## 4. Discussion

Ammonia is one of the most harmful gases produced in rabbit houses, and its high concentration results in intestinal floral imbalance of livestock [14], and thus results in metabolic syndrome and intestinal inflammation [22]. This study showed that intestinal dysfunction and metabolic malnutrition in rabbits is due to ammonia toxicity. Ammonia impairs the structural integrity of cecal tissues and reduce the level of acetate, butyrate and propionate in the cecum of rabbits.

In this study, ADG and ADFI of rabbits in both LAC and HAC groups were significantly lowered compared with CG. The liver index showed a decreasing trend, and the spleen index was significantly decreased in HAC group. Tao et al. [17] found that weight and spleen index in ducks exposed to 10 ppm and 75 ppm ammonia were significantly reduced, and burden of *Phascolarctobacterium* showed significant positive correlation with spleen weight. Other studies have shown that broilers exposed to 25, 50 and 75 ppm ammonia had significantly reduced the body weight of broilers exposed to 0 ppm [20, 21]. Ammonia (70 mg/kg) has reduced the weight of spleen in broilers [29]. In these studies, the decrease in the growth performance of broilers and ducks post ammonia treatment was related to reduction in organ index and change in the structure of intestinal flora and lipid metabolism.

This study showed that HDL, glucose and apoB serum concentrations were increased while that of apoA1 was decreased significantly, indicating liver damage and disrupted lipid metabolism. Glucose, HDL, LDL, apoA1 and apoB are the important indicators of lipid metabolism. ApoA1 is the main apolipoprotein of HDL, and damage to liver results in reduction of apoA1 contents [30]. HDL is the only lipoprotein that transports cholesterol from peripheral tissues to the liver [31]. Tang et al. [32] showed a decreased in the concentration of serum apoA1. Similarly, Sa et al. [21] found that ammonia can significantly increases the level of serum HDL, de novo fat synthesis and cholesterol transportation in broilers. Xing et al. [33] reported that with increase of ammonia concentration, the level of HDL and HDL/LDL ratio in broiler serum was also increased. ApoB is a skeleton protein responsible for lipoprotein synthesis, secretion and transportation. It plays a vital role in the metabolism of LDL and can directly or indirectly affect the body’s abdominal fat deposition and growth [34]. The key function of LDL is transportation of cholesterol to cells throughout the body [35]. Tang et al. [32] found that the concentration of apoB was increased when broilers were exposed to ammonia, indicating that ammonia affect lipid metabolism. In rabbits, low-grade inflammation and fat deposition have been known to negatively impact growth [36]. This study also showed that ammonia disrupts the lipid metabolism of rabbits by affecting the secretion of blood lipids based on hormones related to lipid metabolism, and thus reduces the growth performance.

This study also confirmed vascular degeneration in cecal tissue and mucosal epithelial cells, and edema of lamina propria in ammonia treated groups. The thickness of cecal mucosa and muscle membrane was significantly reduced. Previous study showed that high concentration of ammonia causes intestinal mucosal damage [16], thinning of intestinal wall, shortening of intestinal tract, and shrinking of intestinal villi which are detrimental to intestinal development and nutrient absorption [37]. Intestinal mucosal damage is mainly caused by inflammatory cytokines produced in response to symbiotic bacteria [38, 39]. This study showed that abundance of Clostridium and Proteobacteria in both LAC and HAC groups were increased. Clostridium causes mucosal damage [40] while Proteobacteria has pro-inflammatory properties [41] and its role in intestinal inflammation has been demonstrated in various mouse models of colitis [42]. However, bacterial phenotypes prediction in this study showed that the number of potential pathogenic bacteria in the flora of ammonia treated groups was higher than that in control group, indicating that increase of intestinal pathogenic bacteria has affected intestinal health. Damage to the cecal integrity can change the composition of intestinal microbes. This study showed that Simpson in the HAC group was significantly decreased, indicating that ammonia has reduced the cecal microbial diversity of rabbits, which is consistent with the results obtained by Tao et al. [17] on the laying duck. It has been reported that compared with poorly diverse microbial flora, the intestinal flora is more stable and can improve the health of animals [43]. Drastic changes in microbial species and diversity in the intestinal mucosa may cause microbial imbalance and pathogenesis [44]. The results of this study showed that exposure to high ammonia concentrations significantly reduced the diversity of cecal microflora and increased the burden of pathogenic bacteria. These harmful bacteria cause inflammation and damage to cecal mucosal epithelium which increases with the increase of ammonia concentration.

This study evaluated that Firmicutes, Bacteroidetes and Verrucomicrobia were the dominant phyla in the cecum of rabbits, which is consistent with the results of Huybens et al., Crowley et al., Velasco-Galilea and Chen et al. [45–48]. The burden of Firmicutes and Bacteroides were decreased and increased, respectively in the ammonia treated groups. These results are in agreement with Tao et al. [17], who reported that the burden of Bacteroidetes was significantly higher in ducks exposed to 75 ppm ammonia compared with ducks exposed to 0 and 10 ppm ammonia. Firmicutes contained genes encoding non-starch polysaccharide degrading enzymes which are involved in the breakdown of polysaccharides and use of energy in the intestine [49]. Crowley et al. [46] indicated that shift in the ratio of Firmicutes and Bacteroidetes phyla may affect the digestive efficiency of dietary fibers and the ratio of Firmicutes to Bacteroides were positively correlated with animal growth performance [50]. Therefore, reduction of Firmicutes in the ammonia treated groups in this study may be the reason for the decrease in ADG of rabbits. The functional differences among groups were analyzed through predicted microbial function comparison. It was found that ammonia exposure affected the phospholipid biosynthesis I, glycogen degradation I and phosphatidylglycerol biosynthesis II pathways. Fang et al. [51] found that KOs correlated with energy metabolism and KEGG pathways related to monosaccharide metabolism showed negative correlations with weaning weight (Fang 2019). Studies suggested that gut microbiome with more capacity to consume energy would increase body fat accumulation, which could then hinder growth hormone (IGF-1) secretion resulting in reduced growth performance [52–54]. Previous study has shown that high concentrations of ammonia damage intestinal structure, affect intestinal digestive enzymes level and nutrient transfer of mucosal epithelium, and thus results in reduction of nutrient digestibility in livestock and poultry [46]. In this study, ammonia treatment affected the body energy and lipid metabolism by adjusting the composition of flora, which affects the growth performance of rabbits.

This study evaluated that the concentration of acetate, butyrate and propionate were decreased with ammonia treatment. The burden of *Ruminococcaceae_NK4A214_group* and *Barnesiellaceae_unclassified* were negatively and positively correlated with acetate and propionate, respectively. SCFA is mainly produced by Firmicutes and Bacteroidetes in the posterior segment of intestine [55]. Acetate, butyrate and propionate are the three main SCFA produced by intestinal fermentation, accounting for more than 95% of the intestinal SCFA contents. SCFA absorbed by organs is used as substrates or signaling molecules [56]. SCFA secrete various peptides related to metabolism by binding to G protein-coupled receptors, thereby affecting lipid storage and energy balance. In ammonia treated groups, the burden of *Lachnospiraceae*, *Roseburi* and *CAG-352* and *Barnesiellaceae_unclassified* in Bacteroidetes was significantly reduced, which were related to the hydrolysis of starch and other sugars to produce butyrate and other short-chain fatty acids [57–59]. Butyrate is the main SCFA produced by *Roseburia*/*Eubacterium*. *Lachnospiraceae* produces acetate [60, 61]. *Barnesiellaceae* plays a crucial role in maintaining health status of the body. The bacteria rich in *Barnesiellaceae* prevent blood stream infection (BSI), sarcopenia (PF&S) and other diseases [62, 63]. Compared with obese teenagers, *Barnesiellaceae* is a biomarker of normal-weight adolescent’s microbiota [64–66]. *Barnesiellaceae* is related with the production of SCFA, which was confirmed in this study. Post ammonia treatment, the abundance of *Barnesiellaceae* was significantly reduced which was positively correlated with the production of apoA1 and propionate, while negatively correlated with HDL, LDL and Glu. *Ruminococcaceae* is one of the most abundant genera of Firmicutes, present in the intestinal tract of mammals. As a producer of SCFAs, it is responsible for the degradation of various polysaccharides and fibers related to maintenance of intestinal health [67]. The burden of *Ruminococcaceae_NK4A214_group* was decreased and showed significant positive correlation with TG and HDL, while negative correlation with acetate. Our study showed that ammonia has reduced the concentration of SCFA and affected lipid metabolism, due to reduction in the relative burden of cecal *Barnesiellaceae* and *Ruminococcaceae_NK4A214_group* in the ammonia treated groups. VFA from food fermentation plays critical role in the maintenance of an acidic environment in the intestines [68]. A comparison of the cecal environment between healthy rabbits and those with diarrhea showed that the concentration of acetic acid decreased in the ceca of rabbits with diarrhea [44]. The insufficient amounts of SCFAs have negative impact on inducing the synthesis of insulin like growth factor 1 (IGF-1) which may affect growth performance via IGF-1/ GH axis [68].

## 5. Conclusions

This study revealed the differences in growth performance, lipid metabolism and colonization of intestinal flora among rabbits treated with different ammonia concentrations. Those housed in 10 and 30 ppm of environmental ammonia inhibited growth performance during the 28 days. With an increase of ammonia concentration, lipid metabolism became deteriorated and the number of potential harmful bacteria increased, reducing the abundance of beneficial bacteria, and thus damages structural integrity of cecum and decreases SCFA concentration. These results will help to improve our understanding regarding the possible hidden molecular mechanism involved in the damage of rabbit’s health in harmful gases production.
